# Injury incidence in golf—a systematic review and meta-analysis

**DOI:** 10.1007/s11845-024-03759-6

**Published:** 2024-07-19

**Authors:** Ilari Kuitunen, Ville T. Ponkilainen

**Affiliations:** 1 Institute of Clinical Medicine and Department of Pediatrics, University of Eastern Finland, Kuopio, Finland; 2 Department of Pediatrics, Kuopio University Hospital, Yliopistonranta 1, 70100 Kuopio, Finland; 3 Department of Orthopaedics and Traumatology, Tampere University Hospital, Tampere, Finland; 4 Faculty of Medicine and Health Technologies, Tampere University, Tampere, Finland

**Keywords:** Epidemiology, Golf, Injury, Sports medicine

## Abstract

**Objective:**

There is a lack of comprehensive analysis of injuries in golf per exposure time. Thus, the aim was to report the pooled incidence of injuries in golf.

**Methods:**

We searched PubMed, Scopus, SPORTDiscus, and Web of Science databases in March 2024 for this systematic review and meta-analysis. We included observational studies reporting the number of injuries per exposure time. A random-effects model was used to calculate the pooled injury incidence per 1000 athlete exposures (18 holes of golf) with 95% confidence intervals (CI). Incidences were separately analyzed for men, women, amateurs, professionals, and special athletes.

**Results:**

A total of 999 studies were screened, 29 full texts were assessed, and 7 studies with 269,754 athlete exposures were included. Seven studies assessed the overall incidence of injury, and the pooled estimate was 2.5 per 1000 athlete exposures (CI 0.9–7.5). The incidence was higher in special athletes (21.0, CI 7.7–45.1; one study) than among professionals (8.5, CI 7.6–9.4; one study), or in amateurs (1.3, CI 0.5–4.0; five studies). The injury incidence was 2.6 per 1000 athlete exposures (CI 0.7–9.6; four studies) in women and 1.4 per 1000 athlete exposures (CI 0.4–5.2; three studies) in men. A sensitivity analysis without special athletes had an incidence of 1.9 (CI 0.7–4.9; six studies).

**Conclusion:**

The injury incidence in golf is 2.5 injuries per 1000 athlete exposures (18 holes of golf). Reporting was limited as only one study reported injuries per exposure time in professionals, and in total, only seven studies were found. More research is needed in all levels and age groups to better estimate the injury incidence and associated risk factors in golf.

**Supplementary Information:**

The online version contains supplementary material available at 10.1007/s11845-024-03759-6.

## Introduction

Golf has been among the fastest-growing sports with over 66 million players globally [1]. Due to its nature, golf has been a popular sport for players of all ages and genders. Golf has been found to promote health on many levels [2, 3], for example, it improves cardiovascular health [4], balance [5], and mental health [6]. Golf has a relatively low to moderate injury risk compared to other sports [2, 7].

Professional golfers have higher injury rates than amateur golfers [9]. The most common injuries in professional golfers have been reported to be lumbar spine injuries, followed by wrist/hand injuries [8]. Increased rates of back pain in all golfers have been associated with older age, higher body mass index, and history of back pain [10]. Thus, it is unsurprising that the lower back is the most typical injury, as the swing motion and forces during the rotation move are high [11]. Furthermore, the biomechanics in the golf swing have been shown to cause the swing lead side (meaning the left side in right-handed players and vice versa) to suffer more injuries in the wrist [12], shoulder [13], and knee regions [14]. Interestingly, a study where the hips of professional players were scanned using magnetic imaging showed that the leading hip had more morphological findings than the trail hip [15]. Injury locations and mechanisms have been relatively well studied in golf, but the reporting has been heterogeneous. This led to the publication of a consensus statement in 2020 to guide future studies in regard to conducting and reporting studies of injuries in golf [16].

Previous observational studies have reported varying rates of injuries in golf as the incidence estimated have varied between 0.3 and 0.7 per 1000 h of exposure [4, 7] and 0.2 to 13.4 per 1000 athlete exposures [17, 18]. As the popularity of golf continues to increase and golf has been associated with positive health outcomes, it is important to address the risk of injury. However, there are no previous reviews summarizing the injury incidence in golf per exposure time. Thus, the aim of this systematic review and meta-analysis was to estimate the pooled injury incidence in golf.

## Methods

### Search process

We performed a systematic search initially in December 2022 and updated the search in February 2024 in PubMed, Scopus, SPORTDiscus, and Web of Science databases. The following search phrase was utilized: golf and injur*. Results in the SPORTDiscus were limited to peer-reviewed publications. Additional filters were not used in the search process. The search results were then uploaded to the Covidence software for screening. Two authors performed the screening of abstracts and full texts independently, and the cases of disagreement were solved by mutual decision.

### Inclusion and exclusion criteria

We included all original observational studies that reported the injury incidence in golf per exposure time. We excluded studies that did not report any original data (reviews, editorials, commentaries). Furthermore, conference presentations were excluded, but the official publication was hand-searched, if not retrieved in our initial search. We did not have a priori criteria for injury definition.

### Data extraction

Data was extracted by one author and then verified by the other author to minimize the extracting errors. The following information was extracted from each study: authors, journal, year, study period, participant characteristics, injury definition, follow-up time, number of injuries, exposure time.

### Outcome measures

The main outcome measure was the overall injury incidence while playing golf, reported as injuries per 1000 athlete exposures. Athlete exposure was defined as a full round (18 holes) of golf as suggested by the recent consensus statement regarding the reporting on injuries in golf [16]. As secondary outcomes, we calculated the incidences separately for men, women, amateurs, professionals, and special athletes, which were also suggested in the consensus statement [16]. Special athletes were defined according to the definition of a person to be eligible to participate in the Paralympics or other special athlete events.

### Risk of bias

Risk of bias was assessed according to the Joanna Briggs Institute Critical Appraisal List for Prevalence Studies. The risk of bias was assessed in eight domains and overall [19]. The classification in each domain was yes, unclear, or no. Based on the assessment studies were either included or excluded if serious issues were found.

### Statistics

We have performed the statistical analyses according to the latest version of the *Cochrane* *Handbook for Systematic Reviews of Interventions* [20]. We have performed statistical analysis with R version 4.2.2 software and meta package was used. A generalized linear mixed model was used to calculate pooled incidences per 1000 athlete exposures. Forest plots are presented for all outcomes. The random-effects model was chosen due to expected heterogeneity in the injury definition and study populations. Subgroup analyses (based on gender and level of play) were used to reduce the expected heterogeneity. If the study reported the exposure time as hours, it was then converted to full 18 rounds of golf by dividing the exposure hours by four. Four hours is a rather typical duration of a full round of golf. If the study reported injury incidence and number of injuries, the exposure time was calculated from those.

### Reporting

This systematic review and meta-analysis have been reported according to the preferred reporting items in systematic reviews and meta-analysis (PRISMA) and Implementing Prisma in Exercise, Rehabilitation, Sport medicine and SporTs science (PERSIST) guidelines [21, 22].

### Permissions and registration

This study did not need research permission due to its study design. We registered our protocol to the Prospero database.

## Results

**Fig. 1:**
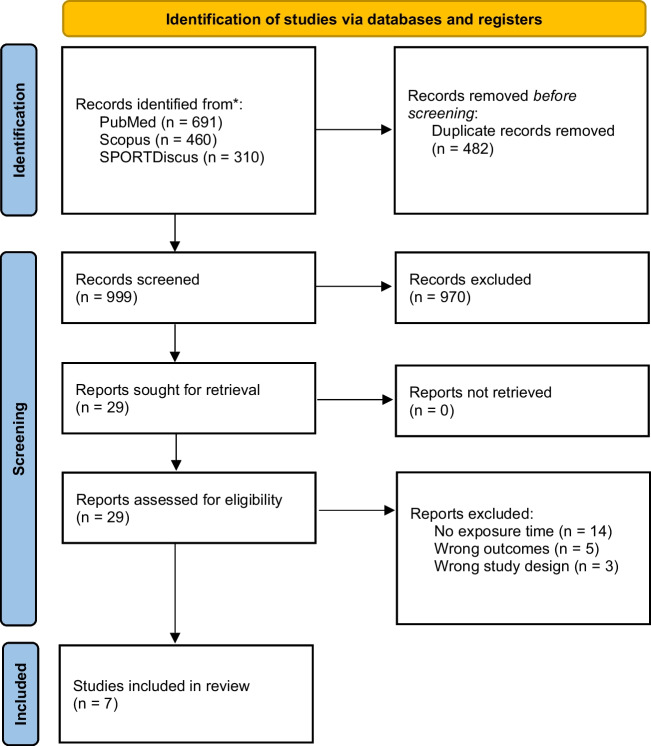
PRISMA flowchart of the review process

We screened 999 abstracts and assessed 29 full reports (Fig. 1). Finally, seven studies were included in the systematic review and meta-analysis [4, 7, 17, 18, 23–25]. Three of the included studies were from Europe, two from the USA, one from Asia, and one from Australia (Table 1). Six were prospective and one study was retrospective. Player handicaps were defined in two of the included studies. Injury definitions had variations between the studies (Table 1).

**Table 1:** Characteristics of the included studies

Study	Country	Study period and duration	Study design	Level of play	Handicap	Gender, sex	Age	Injury definition	Main outcome	Other
Joeng 2018	South Korea	2015 to 2016 (24 motnhs)	Prospective cohort with injury questionnaires	Professional	Not specified but low	Female	22.3 years (SD 3.3)	New or recurring musculoskeletal complaints that occurred in competition or practice	Incidence, location, and types of injuries	Only tournament rounds were included from this study
Kerr 2016	USA	2011 to 2014 (36 months)	Prospective cohort	High school, defined as amateurs in this review	Not specified	Both	Not specified	Occurred as a result of participation in an organized practice or competition and received medical attention by a trainer or physician	Time-loss and non-time-loss injury incidences in high school sports	Study was not designed to focus on golf
McHardy 2007	Australia	2004 to 2005 (12 months)	Prospective survey	Amateurs	Men 17.8 (SD 6.5), women 26.7 (SD 9.2)	Both	59.1 years (SD 12.9)	Any condition sustained during the playing/practicing of golf that stopped play/practice, impeded normal performance, or required medical treatment or medication	Injury incidence and mechanisms	Survey was administered after 12 months and caused a notable recall bias and a likely underestimation to results
Newman 2022	USA	2018 (special Olympic games)	Prospective cohort	Special athletes	Not specified	Both	Not specified	Sprains, strains, and bony injuries recorded and defined by medical volunteers	Injury incidence	Only musculoskeletal injuries were included from this study. This study was not designed to focus only on golf
Parkkari 2000	Finland	Year not specified, 20 weeks	Randomized trial originally, but analyzed as prospective cohort in our review	Amateurs	Not specified	Men	55 years (SD 4)	Any traumatic or overuse injury occurring during a golf game or practice that made the player unable to participate in the following session of golf	Health benefits of golf. Injury incidence as secondary outcome	Study was not designed to analyze injuries in golf and is underpowered for this
Parkkari 2004	Finland	Not specified. 12 months	Prospective cohort	Amateurs	Not specified	Both	Not specified	A new trauma or overuse injury that caused a significant complaint to the subject	Injury incidence	Study was not designed to focus on golf and thus is underpowered
Perron 2016	France	2015	Retrospective cross-sectional survey	Amateurs	< 10	Both	48.5 years (SD 12.8)	Not specified	Injury incidence and locations	Retrospective questionnaire design decreases the validity of the results due to uncontrollable biases

### Risk of bias

Only one study was considered to satisfy all criteria in risk of bias analysis. In the risk of bias analysis, most issues were seen with the validity of the methods used to identify the injury and whether it was measured similarly and reliably to all participants (Table 2). All of the studies utilized appropriate statistical analysis. Three of the included studies had clearly underpowered study sample sizes.

**Table 2:** Risk of bias analyzed according to the Joanna Briggs Institute critical appraisal tool for prevalence studies

Study	Was the sample frame appropriate to address the target population?	Were study participants sampled in an appropriate way?	Was the sample size adequate?	Were the study subjects and the setting described in detail?	Was the data analysis conducted with sufficient coverage of the identified sample?	Were valid methods used for the identification of the condition?	Was the condition measured in a standard, reliable way for all participants?	Was there appropriate statistical analysis?
Joeng 2018	Yes	Yes	Yes	Yes	Yes	Yes	Yes	Yes
Kerr 2016	Yes	Unclear	Yes	No	Yes	Unclear	Unclear	Yes
McHardy 2007	Yes	Yes	Yes	Yes	Yes	No	Unclear	Yes
Newman 2022	Yes	Yes	No	Yes	Yes	Yes	Yes	Yes
Parkkari 2000	Yes	Yes	No	Yes	Yes	Unclear	Unclear	Yes
Parkkari 2004	Yes	Yes	No	Yes	Yes	Unclear	Unclear	Yes
Perron 2016	Yes	Yes	Yes	Yes	Yes	No	No	Yes

### Injury incidences

**Fig. 2:**
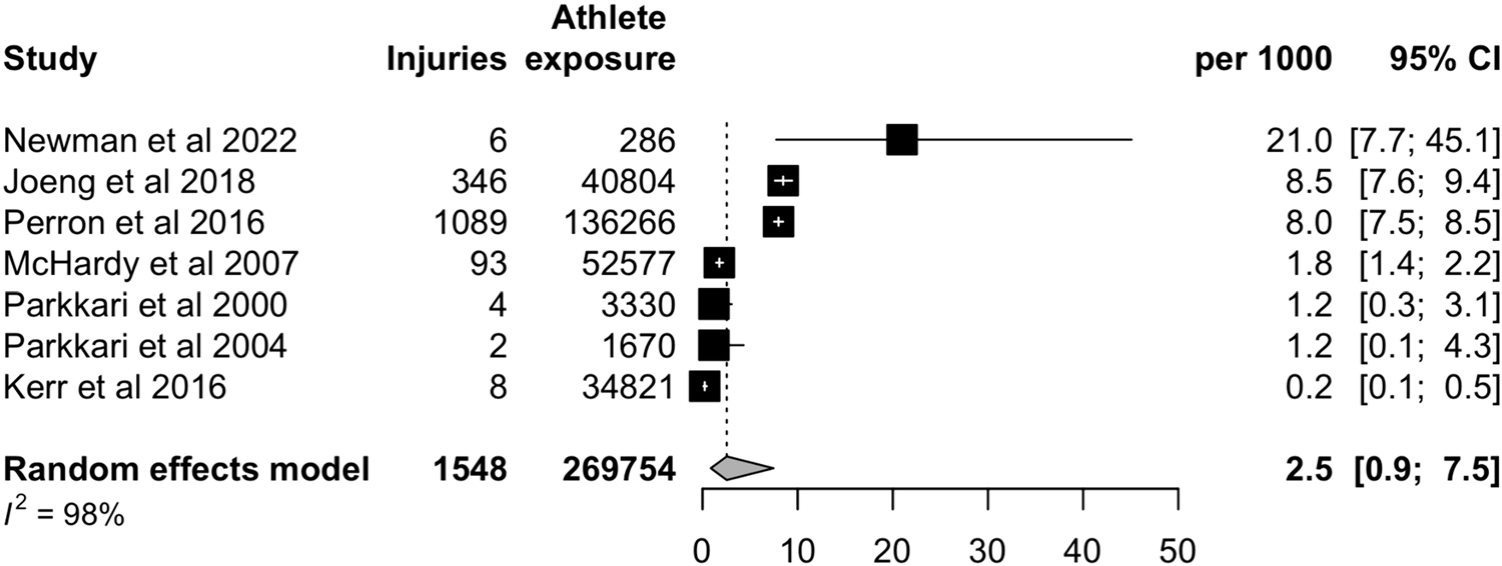
Forest plot of the overall pooled injury incidence per 1000 athlete exposures (full 18 holes of golf)

**Fig. 3:**
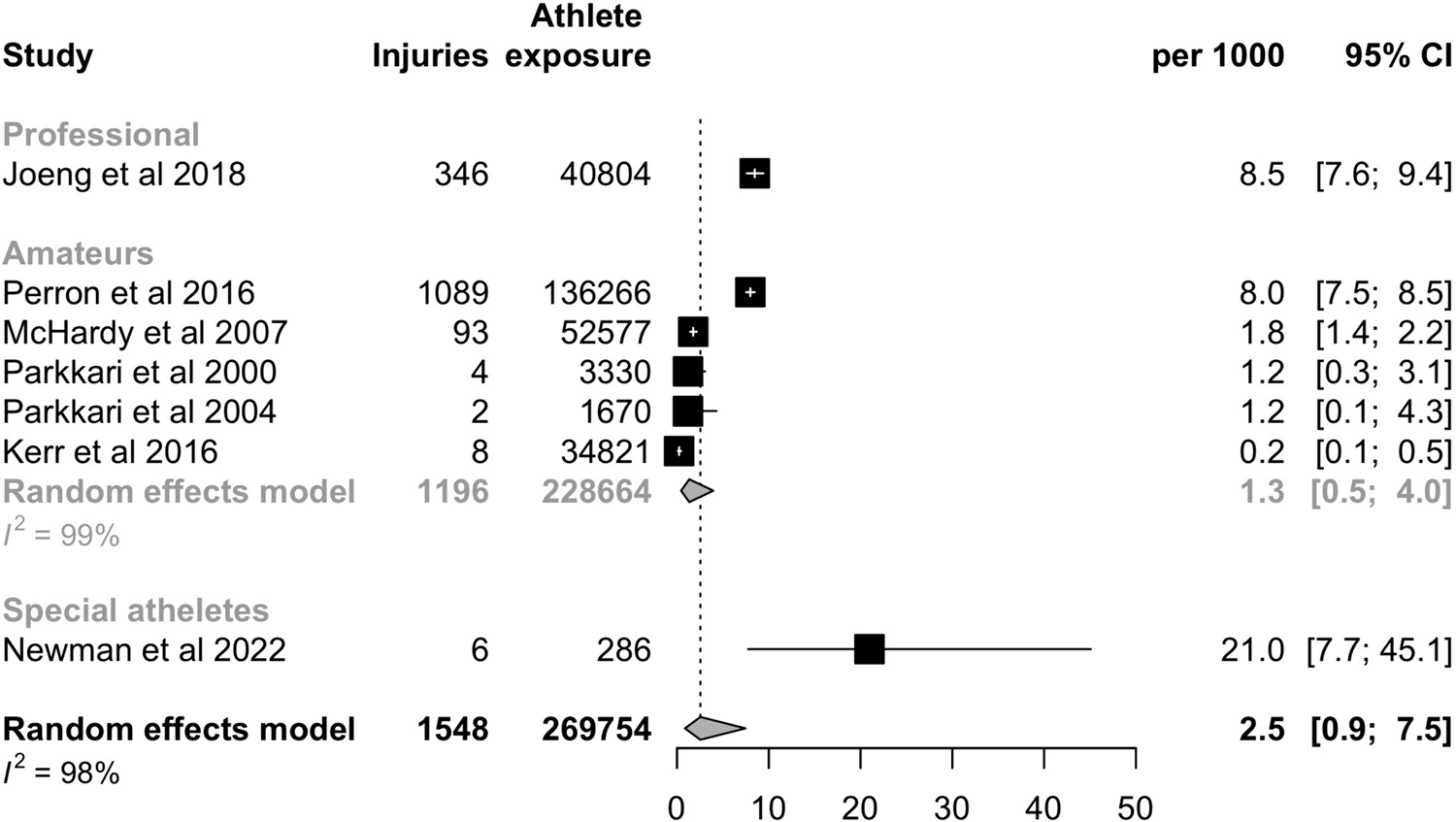
Forest plot of the overall pooled injury incidence per 1000 athlete exposures stratified by the level of play

**Fig. 4:**
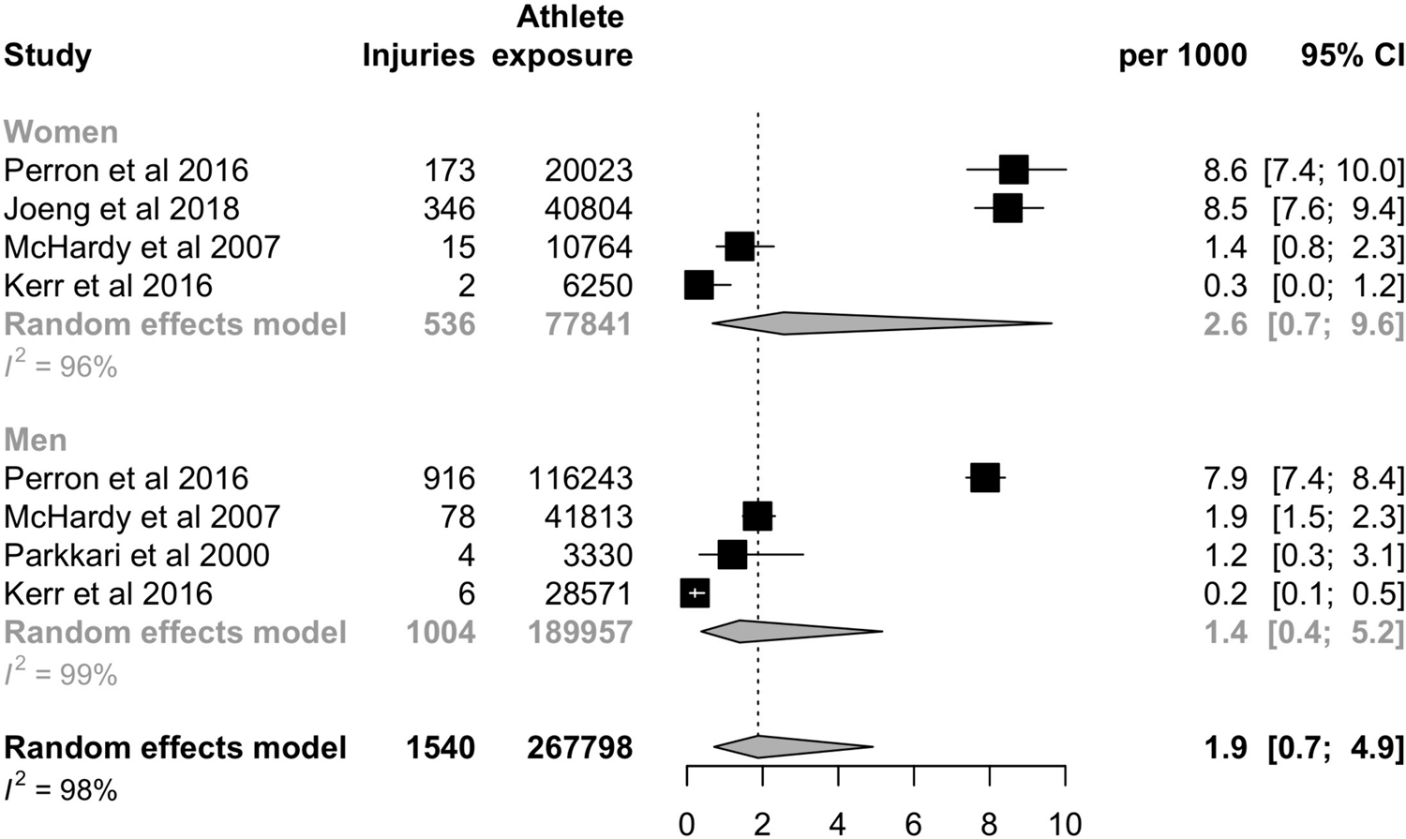
Forest plot of the overall pooled injury incidence per 1000 athlete exposures stratified by sex

Seven studies with 269,754 athlete exposures analyzed the overall injury incidence. The incidence was 2.5 (CI 0.9–7.5) per 1000 athlete exposures (Fig. 2). The highest incidence was observed among special athletes (21.0, CI 7.7–45.1, one study) (Fig. 3). Professionals had a higher incidence (8.5, CI 7.9–9.4, one study) than amateurs (1.3, CI 0.5–4.0, five studies). The injury incidence in women was assessed in four studies and it was 2.6 (CI 0.7–9.6; Fig. 4). Similarly, four studies analyzed men and the injury incidence was 1.4 (CI 0.4–5,2; Fig. 4). A sensitivity analysis without special athletes had an incidence of 1.9 (CI 0.7–4.9; six studies).

## Discussion

We found that the overall injury incidence in golf is 2.5 per 1000 full rounds (18 holes) of golf. The highest injury incidence was observed in special athletes, then professionals, and the lowest incidence was among amateurs. Men and women did not show evidence of a difference in injury incidences. Overall, the injury incidences were relatively low in all categories, which makes golf a safe sport in general.

Based on our knowledge and systematic search, this is the first systematic review to present pooled injury estimates per exposure time in golf. Previous studies have presented the injury epidemiology regarding the types of injuries in golf very well, but statistical synthesis that would have notified exposure time has been lacking. The pooled injury incidence of 2.5 per 1000 full rounds ranks golf among sports with low injury incidence [7]. This injury estimate also turns to 1 injury per 400 full rounds of golf or an injury incidence of 0.6 per 1000 h. Thus, this makes golf a relatively safe sport for everyone, regardless of age and fitness. This is important, as golf has been shown to improve cardiovascular health, mental health, and balance especially in the elderly population. These potential benefits are achievable without an increased injury risk. As golf is a growing sport, it is vital to have reports on the injuries to better guide athletes, trainers, and policymakers in their decisions.

It was unsurprising that professionals had higher injury incidence than amateurs, as this has been reported in other sports (ice hockey, rugby, tennis, etc.) as well [26–28]. A possible explanation for the higher incidence in professionals could be better documentation of injuries, higher intensity of training, and competition. Furthermore, previous reports have described that special athletes have higher injury incidences than professional athletes without disabilities [29, 30]. Males have been reported to have more injuries in team sports than women [31]. Previous reports in individual sports have reported similar injury incidences between men and women [32, 33]. In our current review, we did not find evidence of a difference in golf injuries between men and women.

### Strengths

This systematic review was performed according to the pre-defined protocol, and we did not have protocol deviations. Furthermore, this is the largest and first report to provide the pooled injury incidence estimates in golf. We were able to include one study to focus on special athletes, which can be seen as a strength, as sports research has had minimal reporting in general on disabled participants [34].

### Limitations

A clear limitation is the limited number of publications included in this review as only seven studies were eligible. Furthermore, the injury definitions varied between the studies, and thus, this caused heterogeneity in the estimates. Furthermore, there were issues with the risk of bias assessment as the included studies did not control for possible confounders in their analyses, and the presented injury estimates were not age-adjusted or adjusted with injury history. One of the included studies was a retrospective questionnaire and one was prospective but only one questionnaire about the injuries was fulfilled after 12 months of follow-up; thus, both studies have a high risk of recalling bias, and the presented injury estimates may be underestimates rather than overestimates. Another limitation was the lack of injury mechanism. Furthermore, only two of the studies presented handicaps, which was raised as important background information in the recent consensus statement [16]. A further limitation is that the studies were conducted all in high-income countries and in three regions (North America, Europe, Asia, and Australia). Thus, these results may not be generalizable to minorities or to middle- and low-income countries, as golfers seem to have backgrounds with higher socioeconomic status and educational levels [35].

### Implications for future research

More research is needed in all levels of play and age and genders to have better injury incidence estimates in golf. Future research should better attempt to control possible confounders and present age-adjusted injury estimates. Reporting of injury mechanisms would further strengthen the analyses of injury burden in golf.

### Conclusion

The incidence of injuries while playing golf is 2.5 per 1000 full rounds (18 holes). The highest incidence was observed among special athletes. Professionals have a higher incidence than amateurs. Men and women seem to have similar incidences. Thus, it seems that the injury rate in golf is low, although more and better quality evidence is needed to better inform athletes, trainers, and policymakers.

## Supplementary Information

Below is the link to the electronic supplementary material.

Supplementary file1 (PDF 71.1 KB)

## Data Availability

All data extracted from the included studies are available from the corresponding author.
